# Nanosensors Based on Structural Memory Carbon Nanodots for Ag^+^ Fluorescence Determination

**DOI:** 10.3390/nano11102687

**Published:** 2021-10-12

**Authors:** Xi Zhou, Yufeng Cao, Xinji Zhou, Lina Xu, Daihui Zhang, Chunpeng Wang, Fuxiang Chu, Tao Qian

**Affiliations:** 1 School of Chemistry and Chemical Engineering, Nantong University, Nantong 226019, China; zhouxi@ntu.edu.cn (X.Z.); yufengcao@ntu.edu.cn (Y.C.); 1908110044@stmail.ntu.edu.cn (X.Z.); 2 Institute of Chemical Industry of Forestry Products, Chinese Academy of Forestry, Nanjing 210042, China; xulina072@163.com (L.X.); zdh0824@163.com (D.Z.); wangcpg@163.com (C.W.); 3 Co-Innovation Center of Efficient Processing and Utilization of Forest Resources, Nanjing Forestry University, Nanjing 210037, China

**Keywords:** carbon nanodots, silver ion, fluorescent sensor, structural memory, intracellular imaging

## Abstract

Ag^+^ pollution is of great harm to the human body and environmental biology. Therefore, there is an urgent need to develop inexpensive and accurate detection methods. Herein, lignin-derived structural memory carbon nanodots (C_SM_-dots) with outstanding fluorescence properties were fabricated via a green method. The mild preparation process allowed the C_SM_-dots to remain plentiful phenol, hydroxyl, and methoxy groups, which have a specific interaction with Ag^+^ through the reduction of silver ions. Further, the sulfur atoms doped on C_SM_-dots provided more active sites on their surface and the strong interaction with Ag nanoparticles. The C_SM_-dots can specifically bind Ag^+^, accompanied by a remarkable fluorescence quenching response. This “turn-off” fluorescence behavior was used for Ag^+^ determination in a linear range of 5–290 μM with the detection limit as low as 500 nM. Furthermore, findings showed that this sensing nano-platform was successfully used for Ag^+^ determination in real samples and intracellular imaging, showing great potential in biological and environmental monitoring applications.

## 1. Introduction

In the industrialized world, silver is one of the key ingredients in the field of pharmacy, photography, as well as electrical and aerospace industries due to its attractive properties [1,2,3]. Ag^+^ pollution has become a severe worldwide environmental problem with the associated potential harm to the human body and environmental biology [4]. Thus, it is great important to detect Ag^+^ in water for living and production needs and various analytical strategies have been developed for the quantitative determination of Ag^+^, including atomic absorption spectrometry (AAS) [5], inductively coupled plasma optical emission spectrometry (ICP-OES) [6], inductively coupled plasma mass spectrometry (ICP-MS) [7], and the electrochemical method [8]. However, these approaches suffer from either the need for expensive devices or a tedious sample pre-treatment process, which restrict their application in the field of fast monitoring Ag^+^. Therefore, techniques for the rapid and effective detection of Ag^+^ are urgently demanded.

Over the past decades, optical sensors have been developed and utilized for Ag^+^ determination at different levels [9,10,11]. Among them, the fluorescent sensor is regarded as a powerful analytical strategy for Ag^+^ detecting due to its inherent merits, including easy operability, spatiotemporal resolution, and high sensitivity and selectivity [12,13,14,15]. So far, series of fluorescent molecules have been prepared for Ag^+^ analysis based on Ag^+^-participated chelation or other chemical reactions [16,17,18]. Most reported fluorescent probes generally involved in complex preparation process suffer from biotoxicity and low photostability, and Ag^+^ recognition reactions are susceptible to a responsive environment, which severely limits their application [19,20,21,22,23]. Thus, further exploitation of eco-friendly Ag^+^ sensors with nontoxicity and excellent photostability is still an important challenge for researchers.

Carbon nanodots (C-dots), as one of several new nanomaterials, have drawn impressive attention in the area of bio-labeling, cell-imaging, drug-delivery, and so on [24,25,26,27]. Furthermore, C-dots can also be used as a versatile platform for fluorescence (FL) sensing because of their unique photostability and FL emission, excellent water solubility, low toxicity, and biocompatibility [28,29,30]. Moreover, various techniques, such as electrochemical oxidation, microwave irradiation, arc discharge, and hydrothermal carbonization, have been extensively applied to the preparation of C-dots for the detection of metal ions [31]. Liu and co-workers reported an electrochemical method for the synthesis of C-dots, which showed the potential to monitor Fe(Ⅲ) in the environment [32]. Wang et al. fabricated nitrogen doped C-dots via the hydrothermal treatment of citric acid and amino acid at 180 °C and the FL of products exhibited quenching responses to Hg(Ⅱ) [33]. Gao et al. described a route to prepare nitrogen-rich C-dots through a microwave method, based on which a “turn-off” FL probe for Ag^+^ ion detection was achieved [34]. However, the preparation processes of these C-dots have always been triggered by microwave irradiation, acid treatment, electrochemical, or high-temperature techniques, which restrict the large-scale application of C-dots [35]. In addition, to our knowledge, the selectivity of some reported C-dots for Ag^+^ determination is unsatisfactory due to the interference effect of some other metal ions, including Cu^2+^, Hg^2+^, and Fe^3+^ [28]. Therefore, in respect of the fabrication process and practical application, seeking a simple and green preparation technology towards C-dots for Ag^+^ monitoring with high selectivity and sensitivity has received wide attention.

Lignin, as one of the most abundant renewable resources in the world, consists of multiple polyphenols and methoxy functional groups that can serve as metal ions stabilizing and reducing agents [36]. It should be noted that the methoxy or phenolic hydroxyls groups on the lignin can reduce Ag^+^ to metallic silver nanoparticles (Ag NPs), based on which an excellent selectivity towards silver ion may be realized [37]. Furthermore, the heteroatom on lignin can provide more active sites on their surface and better specific interaction with Ag NPs. However, lignin-based materials for Ag^+^ determination still face some challenges, e.g., low specific surface area or effective functional group concentration [38].

**Scheme 1:**
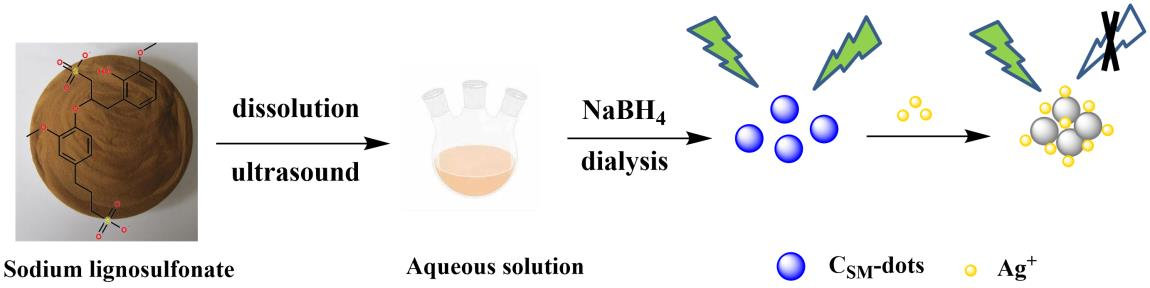
Fabrication process of lignin-derived C_SM_-dots.

It is widely accepted that mild preparation conditions, particularly low pressure and temperatures, can make C-dots reserve functional and structural units of the precursor molecules without chemical reaction [39]. This fabrication strategy of “structural memory”, making full use of the specific recognition functional groups or atoms of the precursors, inspired us to propose lignin-based C-dots for Ag^+^ determination with high selectivity and sensitivity. In this work, a novel sensor towards Ag^+^ was developed based on structural memory C-dots (C_SM_-dots) with sulfur atoms, which were prepared by a facile and green strategy from lignin. The mild preparation process (Scheme 1) allowed the C_SM_-dots to remain plentiful phenol, hydroxyl, and methoxy groups, which have a specific interaction with Ag^+^ through the reduction of silver ions. Moreover, the sulfur atoms doped on C_SM_-dots provided more active sites on their surface and the strong interaction with Ag NPs. Following a demonstration superior FL performance of C_SM_-dots, an eco-friendly Ag^+^ sensor was prepared based on the remarkable FL quenching effect of C_SM_-dots/Ag NPs. Furthermore, this sensing platform was successfully applied for Ag^+^ monitoring in real samples and intracellular imaging without surface chemical modification.

## 2. Materials & Methods

### 2.1. Materials

A variety of metal salts, including AgNO_3_, Ni(NO_3_)_2_, BaCl_2_, ZnCl_2_, CuCl_2_, CaCl_2_, KOH, NaCl, AlCl_3_, FeCl_2_, MgCl_2_, FeCl_3_, MnCl_2_, PbCl_2_, HgCl_2_, Co(NO_3_)_2_, and NaBH_4_ were purchased from Nanjing Chemical Reagent Co., Ltd. (Nanjing, China). Sodium lignosulphonate was obtained from Macklin Biochemical Technology Co., Ltd. (Shanghai, China). All the chemicals were analytical grade and used without further purification. All aqueous solutions were made using deionized water.

### 2.2. Characterization

The transmission electron microscopic (TEM) and high-resolution TEM (HRTEM) images were acquired by a JEM-2100 transmission electron microscopy (JEOL, Tokyo, Japan) at an accelerating voltage of 200 kV. The UV-vis spectra were recorded on a UV-vis absorption spectrophotometer (UV-1800pc) at 800–200 nm. The X-ray photoelectron spectra (XPS) were measured by PHI 500 VersaProbe X-ray photoelectron spectrometer (UlVAC-PHI, Hagisono, Japan). The FT-IR spectra were obtained on an iS10 FT-IR spectrometer at 500–5000 cm^−1^ (Nicolet, Madison, WI, USA). Zeta potential was recorded on Zetasizer Nano ZS instrument (Malvern, Malvern, UK). The FL spectra were acquired by LS SS Perkin Elmer spectrometer (Perkin Elmer, Waltham, MA, USA) with the slit widths of 4 nm for emission and excitation spectra. All the fluorescence spectra were measured from 330 to 700 nm with a quartz cuvette of 1 cm path length.

### 2.3. Preparation of Lignin-Derived C_SM_-dots 

In a typical procedure, sodium lignosulfonate (0.03 g) was dissolved into 15 mL deionized water completely. The solution was treated by ultrasound for 90 min followed with stirring under 1 h. After that, the obtained solution was centrifuged for 30 min to remove precipitates. The supernatant was collected and mixed with NaBH_4_ (0.5 mol/mL) to allow the formation of particles, and then further dialyzed using a dialysis bag (1000 Da cut off) for 12 h. The C_SM_-dots powder was acquired by freeze-drying for further use.

### 2.4. Fluorescence Detection of Ag^+^

A dispersion of the C_SM_-dots was prepared in PBS buffer solution (0.1 M, pH 7.0) with a concentration of 0.05 mg/mL. Hence, 300 μL of C_SM_-dots dispersion, 1200 μL of Ag^+^ solution with different concentration (0–500 μM), and 1500 μL of ultrapure water were mixed in a quartz cuvette. Then, the mixture was moved for FL measurement at 325 nm excitation wavelengths after being incubated for 5 min at room temperature. The same method was applied for a selectivity test, whereby 1200 μL of aqueous solutions containing specific ion (400 μM) were introduced to the above mixture for substituting Ag^+^ solution. 

## 3. Results

### 3.1. Physiochemical Characterization of C_SM_-dots

**Figure 1:**
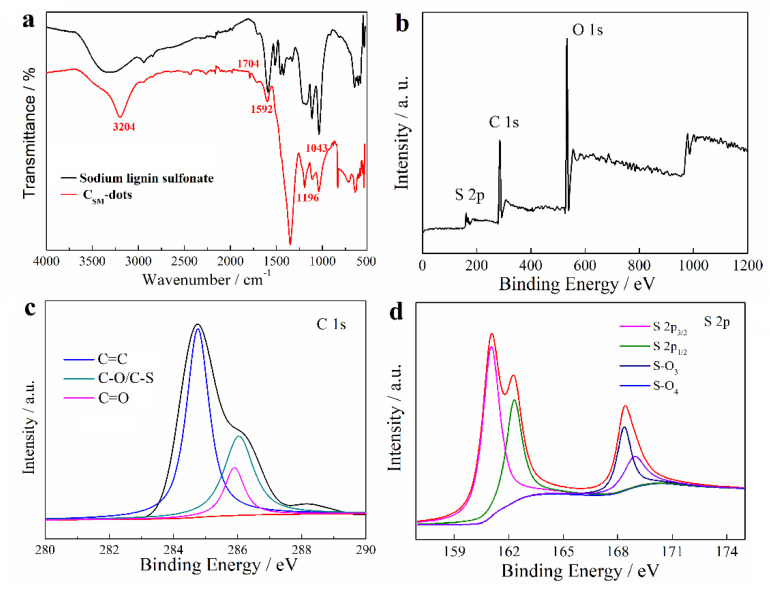
(**a**) FT-IR spectra of lignin and C_SM_-dots. (**b**) XPS spectrum of C_SM_-dots. The high−resolution XPS spectra of C 1s (**c**) and S 2p (**d**) in C_SM_-dots.

During the low temperature preparation process of C_SM_-dots, a considerable portion of functional groups that belong to lignin were retained, which was confirmed by FT-IR (Figure 1a). The peaks at 1704 and 1196 cm^−1^ resulted from the stretching vibration of conjugated C=O bonds and S=O bonds in sulfonic acid groups, respectively. The peaks at 1592 and 1043 cm^−1^ were attributed to the stretching vibrations of C=C bond in the aromatic skeleton and the deformation vibration of C−O−C groups, respectively. The presence of O−H groups was confirmed by the peaks around 3000–3500 cm^−1^ [25]. Moreover, the peak intensity in C_SM_-dots increased, indicating that the addition of NaBH_4_ produced more hydroxyl groups. More convincing details on chemical composition of C_SM_-dots were provided by the XPS spectrum. It was found in the full range XPS spectrum of C_SM_-dots that three peaks at 168.08 eV, 285.08 eV, and 533.08 eV were due to S 2p, C 1s, and O 1s, respectively (Figure 1b). Similar results were found in the XPS spectrum of lignin (Figure S1a), there were three peaks at 167.48 eV (S 2p), 285.75 eV (C 1s), and 532.81 eV (O 1s). Moreover, the high-resolution spectra showed further information on different types of chemical bonds. The C 1s spectrum of lignin (Figure S1b) indicated the presence of C–C (284.5 eV), C=C (283.6 eV), C=O (285.1 eV), and C–O/C–S (286.6 eV). The C 1s spectrum of C_SM_-dots (Figure 1c) indicated the presence of C=C (284.7 eV), C=O (285.8 eV), and C–O/C–S (286.4 eV). The existence of C=C and C=O/C–O peaks further identifies that the C_SM_-dots are enriched with hydroxy and carboxyl functional groups on the surface. The deconvoluted S 2p XPS spectrum (Figure 1d) displayed four peaks at 161.2 eV (S 2p_3/2_), 162.4 eV (S 2p_1/2_), 168.3 eV (S–O_3_), and 168.8 eV (S–O_4_) binding energy, respectively, which clearly indicates that S atoms are present in the structure of C_SM_-dots. Moreover, the spectrum of O 1s (Figure S2a) can be divided into three peaks at 529.5, 531.1, and 533.2 eV, which corresponded to C−O/C−O−C, C=O/S=O, and Ar−OH bonds, respectively [25]. In addition, the morphology and size distribution of the prepared C_SM_-dots were shown in TEM and HRTRM images (Figure S2b). It was found that the C_SM_-dots were well-dispersed, spherical dots with little graphitic lattices, which indicated the amorphous structure of C_SM_-dots. It is because that lignin has an amorphous structure and no severe conditions were applied for the C_SM_-dots fabrication. The size distribution of C_SM_-dots was in a range of 8.2–23.5 nm (based on statistical analysis of more than 200 dots) and almost 72% of the C_SM_-dots were focused on an average diameter between 14.5 and 20.0 nm (Figure S2c). The self-assembly of lignin was largely due to π–π interactions and inter/intramolecular hydrogen bonding [40]. A broad peak at 21.3° in the XRD patterns of C_SM_-dots suggests an amorphous structure (Figure S2d), which was due to the single benzene units and the weak interaction between themselves among the whole amorphous structure of lignin [41]. 

### 3.2. Optical Properties of C_SM_-dots

**Figure 2:**
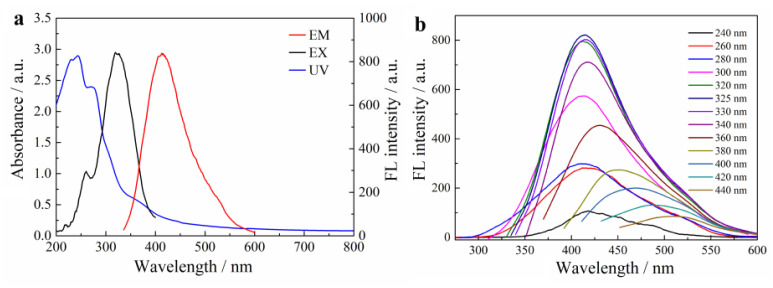
(**a**) The UV-vis absorption spectra, FL excitation and emission spectra of C_SM_-dots. (**b**) The FL emission spectra of C_SM_-dots under different excitation wavelengths.

Optical performance of the C_SM_-dots was characterized by the UV−vis absorption spectrum and the FL spectrum under different excitation wavelengths. Figure 2a shows two obvious absorption peaks at 245 and 273 nm in UV-vis spectrum, which was ascribed to the typical absorptions of the π–π* transition and n–π* transition, respectively [9]. The above results are similar to those of multiple aromatic chromophores that originated from lignin, suggesting the existence of aromatic structures in C_SM_-dots [25]. The FL spectra showed that a maximum emission peak at 415 nm was obtained, which resulted from monitoring the excitation from incident light at 325 nm. Additionally, the C_SM_-dots exhibited an excitation-dependent FL behavior in an excitation wavelengths range of 240–440 nm (Figure 2b). Moreover, the FL quantum yield of C_SM_-dots in the aqueous solution was 13.3% (quinine sulfate as the standard), which is greater than the C-dots derived from biomass-based raw materials without doping [42]. The pH effects on FL intensity of the C_SM_-dots were investigated (Figure S3). This showed a pH-dependence on the FL intensity of the C_SM_-dots, which might be due to pH-sensitive π–π interactions and hydrogen bonding, leading to weakening or strengthening in the FL emission intensity or wavelength. This result provided evidence for the existence of π−π interaction-induced self-assembly in C_SM_-dots. The effects of environment temperature and ionic strength on the FL property were also researched to test the FL stability of as-prepared C_SM_-dots (Figure S4a,b). The FL intensity of the C_SM_-dots showed negligible changes under different temperature and ionic strengths conditions, suggesting that they can be used in different environments.

### 3.3. FL Responses of C_SM_-dots towards Ag^+^ Ions 

Lignin possesses multiple functional groups such as methoxy and polyphenols groups. Earlier reports have confirmed the strong interaction between Ag^+^ and lignin based on its multiple aromatic and methoxy groups functional groups, which can reduce Ag^+^ to Ag NPs [43]. The prepared C_SM_-dots retain functional and structural units of the lignin molecules under the mild preparation conditions. The reductive phenolic hydroxyls or methoxy groups on C_SM_-dots were able to reduce Ag^+^ to Ag^0^ and the sulfur atoms doped on C_SM_-dots provided more active sites on their surface and the strong interaction with Ag NPs. 

**Figure 3:**
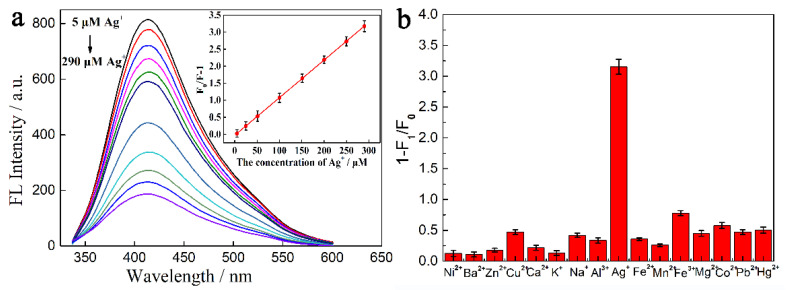
(**a**) The FL emission spectra of C_SM_-dots treated by different Ag^+^ concentrations (from 5 to 290 μM). The inset is the relationship between the *F*_0_/*F* value and Ag^+^ concentrations. (**b**) FL responses of C_SM_-dots in the presence of different metal ions (concentration: 400 μM). *F*_0_ and *F* are the FL intensities in the absence and presence of metal ions, respectively.

The FL responses of C_SM_-dots towards Ag^+^ were demonstrated in Figure 3a. It was found that the FL intensity of the C_SM_-dots at 415 nm decreased with the increasing concentrations of Ag^+^. The relationships between the FL intensity and Ag^+^ concentration are described by the Stern–Volmer equation [27]:

*F*_0_/*F* = 1 + *K*_SV_*C*_Ag+_ (1)

where *F*_0_ and *F* are the FL intensities in the absence and presence of Ag^+^, respectively, *K*_SV_ is the quenching constant of Ag^+^, and *C*_Ag+_ is Ag^+^ concentration. As shown in the inset of Figure 3a, an admirable linear FL response of C_SM_-dots can be detected towards Ag^+^, which was in a concentration range of 5–290 µM. A high linear correlation coefficient (R^2^ = 0.994) was achieved with the limit of detection (LOD) as low as 500 nM (S/N = 3). Moreover, the FL response experiments towards other interferences (Ni^2+^, Ba^2+^, Zn^2+^, Cu^2+^, Ca^2+^, K^+^, Na^+^, Al^3+^, Fe^2+^, Mg^2+^, Fe^3+^, Mn^2+^, Pb^2+^, Co^2+^ and Hg^2+^) were carried out under the same situation (Figure 3b). The addition of the above potential interfering substances only brought about negligible changes in the FL intensity. To further certify the selectivity of C_SM_-dots, the quenching efficiency (1−*F*/*F*_0_) of Ag^+^ coexisting with other different ions in the system was measured (Figure S5). Although the interference concentration is ten times higher than that of Ag^+^, no significant changes in quenching efficiency were found. The results of selectivity tests reflected the fact that the C_SM_-dots had high selectivity for Ag^+^ sensing, which provided the precondition for developing a multi-functional sensing system.

A comparison of different methods for Ag^+^ determination is summarized in Table S1, which shows the superiority of the proposed method in linear range and LOD. Furthermore, a static quenching process was obtained by the minor FL lifetime change (Figure S6a). The average FL lifetime of C_SM_-dots was transferred from 8.67 ns to 7.98 ns after the addition of Ag^+^, which probably resulted from the formation of a quite stable nonradioactive FL complex between C_SM_-dots and Ag^+^ [37]. TEM coupled Zeta potential characterization confirmed the evidence of an aggregation induced quenching mechanism. When 500 µM Ag^+^ was added to the C_SM_-dots solution with standing overnight, the TEM image displayed the obvious aggregation behavior (Figure S6b). Moreover, the changes in zeta potential values of C_SM_-dots from −38.45 to 3.50 mV after the addition of excess Ag^+^, further suggested the aggregation effect between the lignin-derived C_SM_-dots and Ag^+^. To further verify the binding mechanism of C-dots towards Ag^+^, the XPS spectroscopy (Figure S7a) was used to characterize the elemental composition of C_SM_-dots/Ag NPs. Compared with the full scan spectrum of C_SM_-dots, the XPS spectrum of C_SM_-dots/Ag NPs had two new peaks at 368.4 eV (Ag 3d_5/2_) and 374.3 eV (Ag 3d_3/2_), suggesting the successful reduction of Ag^+^ into Ag NPs (Figure S7b). Besides, the chemical bonding of C, O, and S was characterized by deconvoluted high-resolution C 1s, O 1s, and S 2p spectra. The S 2p spectra of C_SM_-dots/Ag NPs indicated three bands at 168.2 eV (-C-SO_x_-), 169.4 eV (–C–S–C–), and 167.9 eV (Ag-S) binding energy (Figure S7c), which demonstrated the participation of the S atom in the Ag–S bond. Moreover, it resulted in the successful formation of the C_SM_-dots/Ag NPs. The deconvoluted O 1s spectra showed three types of O atoms with peaks at 531.6 eV (C–OH), 532.2 eV (C=O/S=O), and 530.8 eV (Ag–O) (Figure S7d), which indicated the interaction of Ag NPs with oxygen-containing groups of C_SM_-dots [25].

### 3.4. Cytotoxicity Test

**Figure 4:**
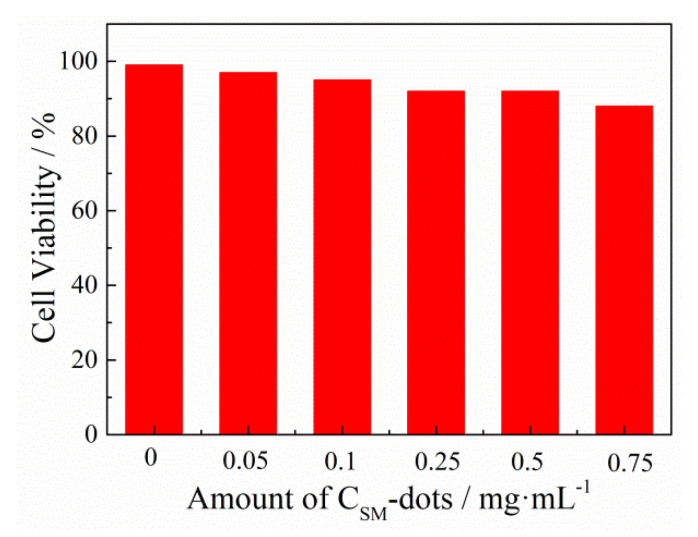
HeLa cell viability after incubation with different concentrations of C_SM_-dots for 24 h.

Biomass-derived C-dots have intrinsic biocompatibility and low toxicity, which provides advantageous conditions for their application in biological fields. Before biological application, the cytotoxicity experiments were carried out using the MTT method, in which the viability of HeLa cells treated with C_SM_-dots was evaluated. It was found in the cytotoxicity experiments that the viability of HeLa cell was shown to be more than 88%, even at a concentration of 0.75 mg/mL, showing excellent biocompatibility and great promise for cellular imaging (Figure 4).

### 3.5. Practical Assay in Real Sample and Cellular Imaging

To confirm the superiority of the approach in real sample, the C_SM_-dots were employed to detect Ag^+^ in tap water samples via the standard addition method. Different concentrations of Ag^+^ were added into tap water samples in the presence of C_SM_-dots. As shown in Table 1, recoveries between 90.7% and 106.3% with relative standard deviation (RSD) lower than 4.5% were achieved at each Ag^+^ concentration. These consequences suggest the reliability and feasibility of the C_SM_-dots for Ag^+^ monitoring in practical sample analysis. In addition, to further estimate the biologic applications of the C_SM_-dots, cell imaging experiments were carried out to demonstrate their feasibility in biological applications. As shown in Figure S8, an intracellular district displayed a remarkable blue emission. Moreover, there was no considerable alteration in the morphology of the HeLa cell, which proved the low cytotoxicity of the C_SM_-dots. All results demonstrated that the prepared nanodots could be used for effectively labeling live cells.

## 4. Conclusions

In summary, a green, economical fabrication strategy for structural memory C_SM_-dots was developed using sodium lignosulphonate as precursors. The as-prepared C_SM_-dots with high FL performance were successfully utilized as a sensing platform for Ag^+^ determination. Due to the aggregation induced quenching effect of Ag^+^ towards C_SM_-dots, the proposed approach offers a rapid and effective tactic for Ag^+^ determination with excellent sensitivity and selectivity. More importantly, the designed sensor was successfully applied for Ag^+^ monitoring in real samples and cellular imaging, demonstrating its potential in practical applications. Therefore, we believed that this method might provide a new insight for the exploitation of multiple functional sensing platforms for biological and environmental applications.

## Figures and Tables

**Table 1:** Determination of Ag^+^ in real samples.

Analyte	Spike (μM)	Found (μM)	Recovery (%)	RSD (n = 3, %)
Ag^+^	50.00	45.35	90.7	2.3
	100.00	96.4	96.4	3.7
	150.00	159.45	106.3	4.5
	250.00	263	105.2	4.4

## Data Availability

The data presented in this study are available on request from the corresponding author.

## Supplementary Materials

The following are available online at https://www.mdpi.com/article/10.3390/nano11102687/s1, Methodology of cytotoxicity test and cellular imaging analysis, Figure S1. (a) The XPS spectra of lignin. (b) The high-resolution XPS spectra of C 1s in lignin. Figure S2. (a) The high-resolution XPS spectra of O 1s in C_SM_-dots. (b) TEM image of C_SM_-dots (The inset is HRTEM image of C_SM_-dots). (c) Size distribution of C_SM_-dots. (d) XRD pattern of CSM-dots. Figure S3. The effect of different pH value on the FL performance of C_SM_-dots. Figure S4. FL intensity variation of the C_SM_-dots as a function of temperature (a) and concentrations of NaCl (b). Figure S5. The selectivity for the detection of Ag+ by C_SM_-dots in the presence of other metal ions. The concentrations of the interference ions were 4000 µM, which is ten time higher than that of Ag^+^. Figure S6. (a) FL decay spectra of C_SM_-dots and C_SM_-dots/Ag^+^ system. (b) The TEM image of C_SM_-dots /Ag^+^ composites. Figure S7. XPS spectrum of C_SM_-dots/ Ag NPs (a). The high-resolution XPS spectra of Ag 3d (b), S 2p (c) and O 1s (d) in C_SM_-dots/ Ag NPs. Figure S8. The fluorescence microscopy images of HeLa cells treated with C_SM_-dots, (a) the bright-field images, (b) the fluorescent images, (c) the merged images of (a) and (b). Table S1. Comparison of the reported probe for Ag^+^ determination. References [44,45,46,47,48] were cited in Supplementary Materials.
