# Supplementary material for: Nanosensors Based on Structural Memory Carbon Nanodots for Ag+ Fluorescence Determination
Source: Nanomaterials (Basel). 2021 Oct 12;11(10):2687. doi: 10.3390/nano11102687 (PMC8537853; doi:10.3390/nano11102687)
Supplement: Supplementary file 1 [file nanomaterials-11-02687-s001.zip › nanomaterials-1339271-supplementary.pdf]

nanomaterials

MDPI

Supplementary Materials

Nanosensors Based on Structural Memory Carbon Nanodots for Ag^+ Fluorescence Determination

Xi Zhou [1,2,3], Yufeng Cao [1], Xinji Zhou [1], Lina Xu [2,3], Daihui Zhang [2,3], Chunpeng Wang [2,3], Fuxiang Chu [2,3,*] and Tao Qian [1,*]

[1] School of Chemistry and Chemical Engineering, Nantong University, Nantong 226019, China; zhouxi@ntu.edu.cn (X.Z.); yufengcao@ntu.edu.cn (Y.C.); 2839376833@qq.com (X.Z.)
[2] Institute of Chemical Industry of Forestry Products, Chinese Academy of Forestry, Nanjing 210042, China; xulina072@163.com (L.X.); zdh0824@163.com (D.Z.); wangcpg@163.com (C.W.)
[3] Co-Innovation Center of Efficient Processing and Utilization of Forest Resources, Nanjing Forestry University, Nanjing 210037, China

*Correspondence: chufxg@163.com (F.C.); qiantao@ntu.edu.com (T.Q.)

Cytotoxicity test: Human uterine cancer cell (HeLa) was seeded into 96-well plates with Dulbecco's modified Eagle's medium (DMEM) for 24 h at a density of 10^4 cells/150 μL in an incubator (37 °C, 5% CO_2). And the culture medium was replaced DMEM containing C_{SM}-dots of different concentrations (0, 0.05, 0.1, 0.25, 0.5, 0.75 mg/mL) for another 24 h. Then, the cells were washed by 20 mL PBS buffer (pH 7.4) and incubated with MTT solution (5 mg/mL) for 4 h. After that, the culture medium was removed, followed by the addition of 150 μL DMSO and shaken for 10 min at room temperature. The optical density (OD) was measured by a microplate reader (ELx800, Biotek, USA) at 490 nm. The cell viability was estimated according to the following equation:

$$\text{Cell viability (\%)} = (OD_{treated} / OD_{control}) \times 100\%$$

where $OD_{treated}$ was obtained in the presence of LGQDs, and $OD_{control}$ was obtained in the absence of C_{SM}-dots.

Cellular imaging analysis: The HeLa was first cultured in DMEM containing 10% fetal bovine serum (FBS) and 1% penicillin/streptomycin at 37 °C in 5% CO_2 for 12 h, followed by incubation with C_{SM}-dots (0.5 mg mL^{-1}) for another 6 h. Then, the cells were washed with Dulbecco's phosphate buffer saline (DPBS) three times (1.0 mL each) to remove the C_{SM}-dots. The Hela cells were harvest by fixed with 4% paraformaldehyde solution in DPBS at 4 °C for 30 min. The cellular imaging of the treated cells was performed on a Nikon Eclipse 90i microscope equipped with the Cool SNaP HQ2 CCD camera (Photometrics, AZ).

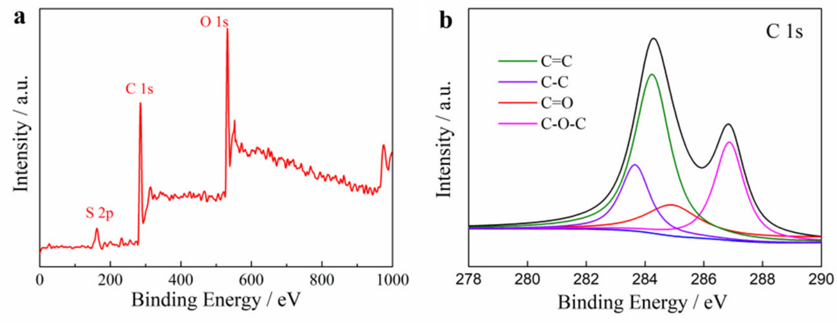

Figure S1. (**a**) The XPS spectra of lignin. (**b**) The high-resolution XPS spectra of C 1s in lignin.

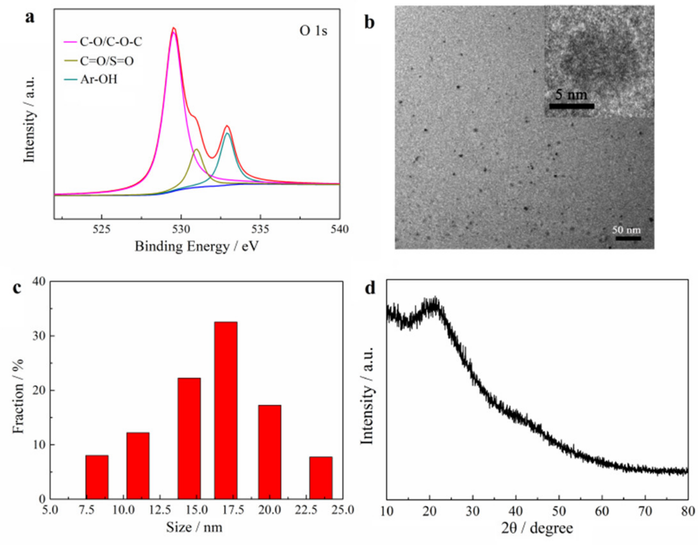

Figure S2. (**a**) The high-resolution XPS spectra of O 1s in C_{SM}-dots. (**b**) TEM image of C_{SM}-dots (The inset is HRTEM image of C_{SM}-dots). (**c**) Size distribution of C_{SM}-dots. (**d**) XRD pattern of C_{SM}-dots.

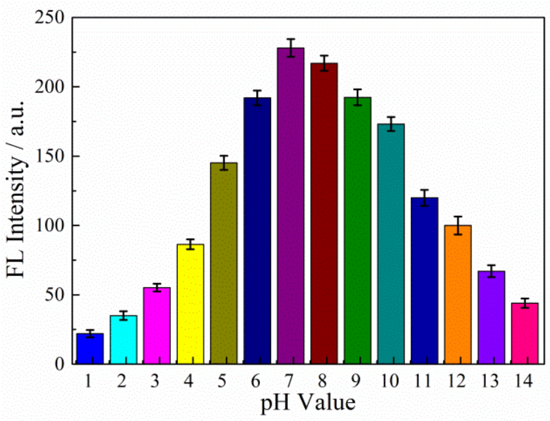

Figure S3. The effect of different pH value on the FL performance of C_{SM}-dots.

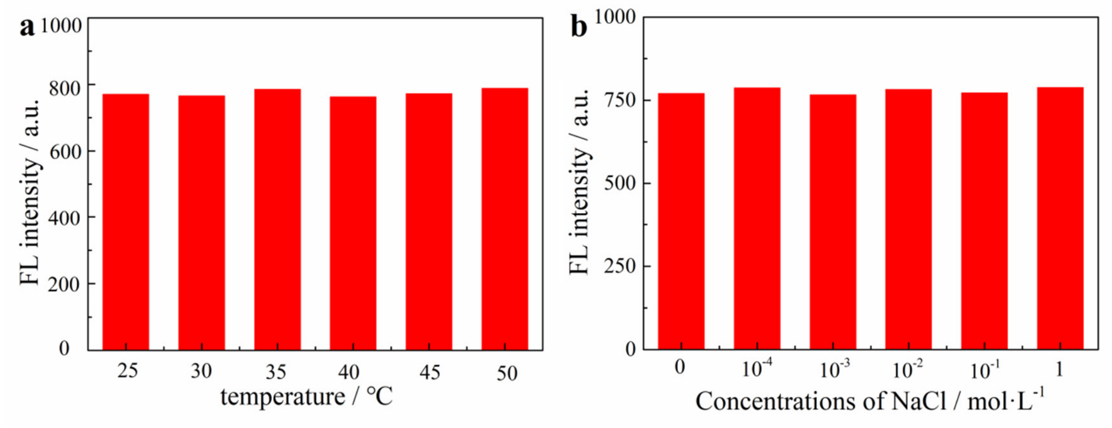

Figure S4. FL intensity variation of the C_{SM}-dots as a function of temperature (**a**) and concentrations of NaCl (**b**).

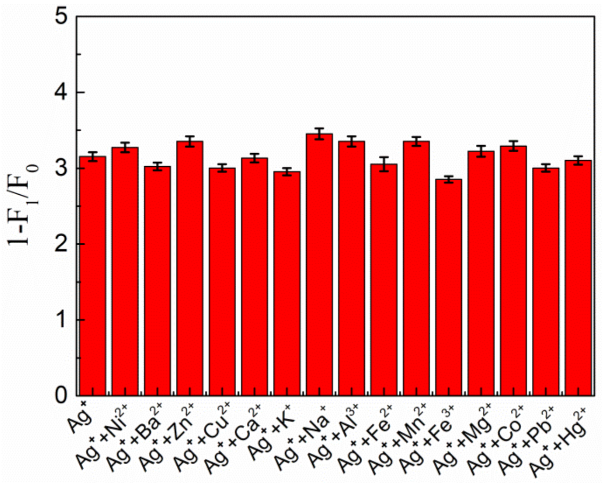

Figure S5. The selectivity for the detection of Ag^+ by C_{SM}-dots in the presence of other metal ions. The concentrations of the interference ions were 4000 μM, which is ten time higher than that of Ag^+.

Table S1. Comparison of the reported probe for Ag^+ determination.

Precursor	Linear range (μM)	Detect limit (nM)	Ref.
Pyrrolo[2,1-a]isoquinoline	0-30	600	[1]
1,2,4-Triaminobenzene	0-30	660	[2]
Polyurethane foam	0-6	2800	[3]
azidoimidizole	0-20	480	[4]
Bis(5,6-dimethylbenzimidazole)	0-100	423	[5]
Lignin	5-290	500	This work

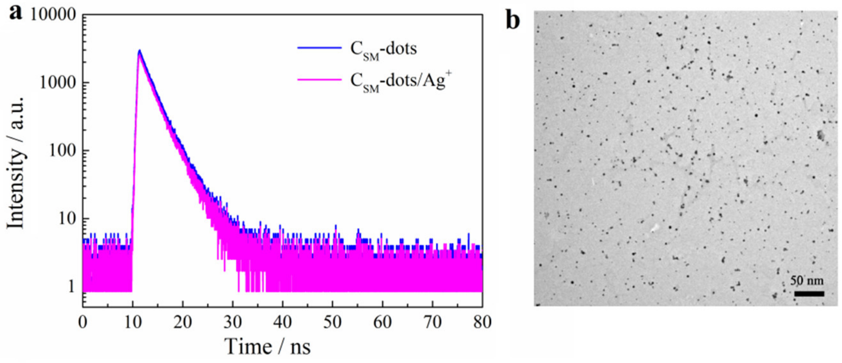

Figure S6. (**a**) FL decay spectra of C_{SM}-dots and C_{SM}-dots/Ag^+ system. (**b**) The TEM image of C_{SM}-dots /Ag^+ composites.

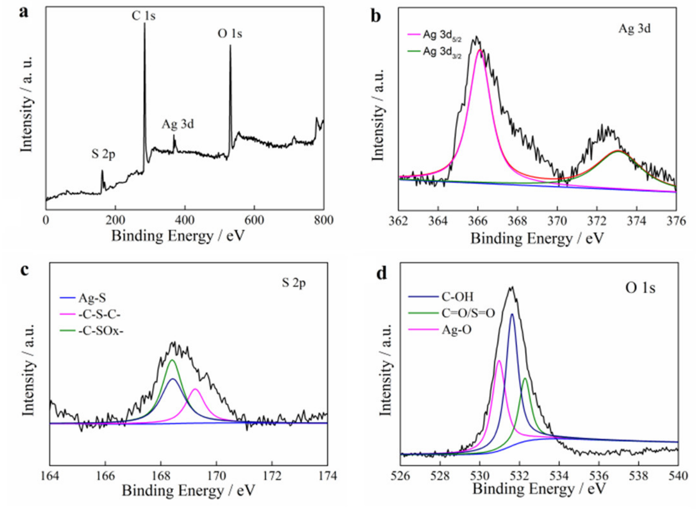

Figure S7. XPS spectrum of C_{SM}-dots/ Ag NPs (**a**). The high-resolution XPS spectra of Ag 3d (**b**), S 2p (**c**) and O 1s (**d**) in C_{SM}-dots/ Ag NPs.

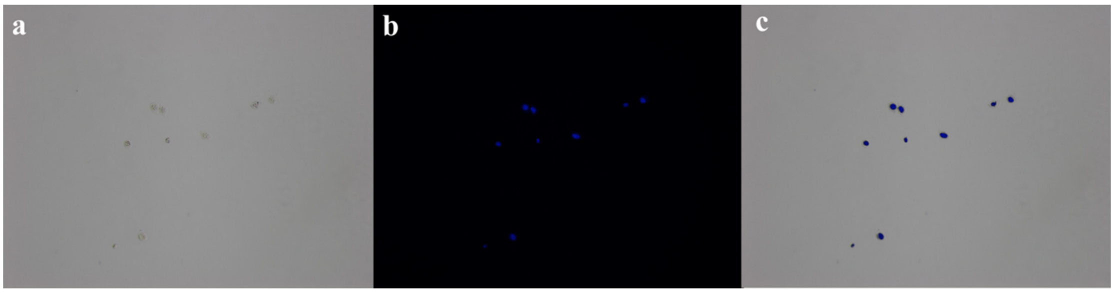

Figure S8. The fluorescence microscopy images of HeLa cells treated with C_{SM}-dots, (**a**) the bright-field images, (**b**) the fluorescent images, (**c**) the merged images of (**a**) and (**b**).

References

1. Jiang, Y.L.; Kong, W.W.; Shen, Y.M.; Wang, B.X. Two fluorescence turn-on chemosensors based on pyrrolo[2,1-a]isoquinoline for detection of Ag^{+} in aqueous solution. *Tetrahedron* **2015**, *71*, 5584, doi: 10.1016/j.tet.2015.06.055.
2. Lu, Z.F.; Su, T.T.; Feng, Y.T.; Jiang, S.Q.; Zhou, C.X.; Hong, P.Z.; Sun, S.L.; Li, C.Y. Potential application of nitrogen-doped carbon quantum dots synthesized by a solvothermal method for detecting silver ions in food packaging. *Int. J. Environ. Res. Public Health* **2019**, *16*, 2518, doi: 10.3390/ijerph16142518.
3. Shen, C.; Ge, S.Y.; Pang, Y.Y.; Xi, F.N.; Liu, J.Y.; Dong, X.P.; Chen, P. Facile and scalable preparation of highly luminescent N, S co-doped graphene quantum dots and their application for parallel detection of multiple metal ions. *J. Mater. Chem. B* **2017**, *5*, 6593, doi; 10.1039/C7TB00506G.
4. Wu, Z.S.; Feng, M.K.; Chen, X.X.; Tang, X.J. N-dots as a photoluminescent probe for the rapid and selective detection of Hg^{2+} and Ag^{+} in aqueous solution. *J. Mater. Chem. B* **2016**, *4*, 2086, doi; 10.1039/C5TB02628H.
5. Wu, Y.C.; Jiang, K.; Luo, S.H.; Cao, L.; Wu, H.Q.; Wang, Z.Y. Novel dual-functional fluorescent sensors based on bis(5,6-dimethylbenzimidazole) derivatives for distinguishing of Ag^{+} and Fe^{3+} in semi-aqueous medium. *Spectrochim. Acta A* **2019**, *206*, 632, doi: 10.1016/j.saa.2018.05.069.
